# The Phenolic Signature of *Psidium cattleianum* Fruits and Leaves Modulates TRPV1 and TRPA1 Transient Receptor Potential Channels: A Metabolomics, In Vitro, and In Silico Study

**DOI:** 10.1002/fsn3.70075

**Published:** 2025-03-24

**Authors:** Leilei Zhang, Fabio Arturo Iannotti, Fatema R. Saber, Reem K. Arafa, Aniello Schiano Moriello, Rasha A. Rasle, Anton Soria‐Lopez, Sara G. Abd EL‐Gawwad, Gabriele Rocchetti, Paz Otero, Łukasz Kulinowski, Krystyna Skalicka‐Woźniak, Luigi Lucini, Jesus Simal‐Gandara

**Affiliations:** ^1^ Department for Sustainable Food Process Università Cattolica del Sacro Cuore Piacenza Italy; ^2^ Institute of Biomolecular Chemistry (ICB); National Research Council (CNR) Pozzuoli Italy; ^3^ Pharmacognosy Department, Faculty of Pharmacy Cairo University Cairo Egypt; ^4^ Drug Design and Discovery Lab Helmy Institute for Medical Sciences, Zewail City of Science and Technology Giza Egypt; ^5^ Biomedical Sciences Program University of Science and Technology, Zewail City of Science and Technology Giza Egypt; ^6^ Department of Physical Chemistry, Faculty of Sciences Universidade de Vigo Ourense Spain; ^7^ Department of Animal Science, Food and Nutrition Università Cattolica del Sacro Cuore Piacenza Italy; ^8^ Analytical Chemistry and Food Science Department, Faculty of Science Nutrition and Bromatology Group Ourense Spain; ^9^ Department of Natural Products Chemistry Medical University of Lublin Lublin Poland; ^10^ CISPAC Fontan Building, City of Culture Santiago de Compostela Spain

**Keywords:** metabolomics, molecular docking, myrtaceae, phenolic profiling, *Psidium cattleianum*, TRP channels

## Abstract

Although *Psidium cattleianum* (strawberry guava, Myrtaceae) is known for its anti‐inflammatory, antioxidant, antimicrobial, and antidiabetic properties, its phytochemical profile and associated bioactivities remain largely underexplored. This study employed UHPLC‐QTOF‐HRMS for untargeted phenolic profiling of leaf and fruit extracts from *P. cattleianum* , followed by semi‐quantification of phenolic subclasses and multivariate data analysis. Four hundred sixty‐nine metabolites, including various phenolic subclasses—predominantly flavonoids and phenolic acids were— identified and annotated. Using HEK‐293 cells stably transfected with TRPA1 or TRPV1 cation channels, it was found that both leaf and fruit extracts activate and rapidly desensitize TRPA1 in a concentration‐dependent manner (EC_50_ 18 and 30 μg/mL; IC_50_ 60 and 47 μg/mL, respectively). Additionally, molecular docking analysis provided deeper insights into the interactions between *P. cattleianum* phytochemicals and the TRPA1 cation channel, identifying theaflavin 3,3'‐*O*‐digallate as the phenolic compound with the highest affinity (S score of −9.27 Kcal/mol). Interestingly, except for theaflavin 3,3'‐*O*‐digallate, compounds enriched in the leaf extract exhibited weaker binding interactions and lower S scores (approximately −7 Kcal/mol) compared to those enriched in the fruit extract. Also, a 100 ns molecular dynamics study of theaflavin 3,3'‐*O*‐digallate with TRAP1 demonstrated high binding stability of the complex. Overall, this study offers valuable insights into the phytochemical characteristics of *P. cattleianum* extracts and reveals their mechanism of action through affinity for the TRPA1 cation channel‐receptors.

## Graphical abstract

An untargeted metabolomics study was performed to find the encompassed phytochemicals in the fruit and leaf extract of *Psidium cattleianum.* This enabled the annotation of 469 compounds belonging to different phenolic subclasses, with a predominance of flavonoids and phenolics. This is the first study investigating the effect of *Psidium* extracts on TRPA1 and TRPV1 channels. The fruit extract exhibited significant efficacy against TRPA1 channels, and molecular docking further augmented the biological interactions with the receptors, which supports our molecular findings.

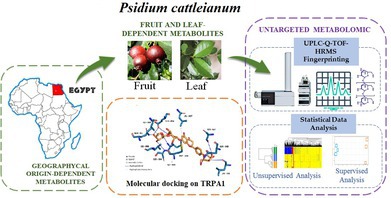

## 1 Introduction

The genus *Psidium* belongs to the family Myrtaceae and is native to Central and South America, from Mexico to Peru (Angulo‐López et al. 2021). However, it is now naturalized in tropical and subtropical zones of Europe, Asia, and Africa due to its ease of adaptation (Díaz‐de‐cerio et al. 2016; Ryu et al. 2021; Ugboko et al. 2020). In addition, these plants can survive constant abiotic stresses, contributing to broad diversification and geographical distribution (Campos et al. 2021).

*Psidium* genus comprises approximately 266 species (Campos et al. 2021), from which *Psidium guajava* L. (common guava) is the most cultivated and studied for its edible fruit (Proença et al. 2022). The common guava has a round‐oval shape, and its skin is green‐yellow, with the pulp varying in color from light yellow to white, pink, or salmon, depending on the specific variety (Mccook‐Russell et al. 2012).

Importantly, *Psidium* spp. are used worldwide to treat symptoms of many diseases because of their pharmaceutical potential and biological activities (Campos et al. 2021; Gutiérrez 2008). Their seeds, roots, leaves, bark, flowers, and fruits are used in traditional medicine, especially as infusions and decoctions for oral and topical use (Naseer et al. 2018). Besides its antioxidant, antimicrobial, anti‐inflammatory, and antidiabetic properties (Schulz et al. 2020; Scur et al. 2016), the additional medicinal uses of *Psidium* spp. include the treatment of gastrointestinal and respiratory diseases (Lozoya et al. 2002; Meles et al. 2021) as well as illnesses caused by pathogenic microorganisms and disorders related to organ malfunction (Morais‐Braga et al. 2016). Phytochemical analysis of *P. guajava* confirmed the presence of phenolics as major compounds, as well as meroterpenoids and essential oils, which were also widely investigated (Julio et al. 2018; Kumar et al. 2021; Qin et al. 2017; Wang et al. 2021).

*Psidium cattleianum* Sabine ( *P. cattleianum* ), also known as strawberry guava, is less popular and not extensively studied when compared to *P. guajava* . *Psidium cattleianum* has two widely known varieties, those with yellow and red fruit cultivars (Luximon‐ramma et al. 2003). This plant is native to Brazil but is currently present in tropical and subtropical climate regions such as Hawaii, the Caribbean islands, Cuba, and Egypt (Campos et al. 2021). It is often described as more aromatic and sweeter than *P. guajava* , with a flavor resembling strawberries (Mccook‐Russell et al. 2012). According to a comparative study of both species, *P. cattleianum* and *P. guajava* , it was shown that the first species was found to be a better source of nutrients, with a greater content of phenolics and vitamin C and with higher antioxidant and antimicrobial activities, but with minor anti‐inflammatory activity (Mccook‐Russell et al. 2012).

Generally, *P. cattleianum* fruits are harvested from native vegetation or small domestic orchards and sometimes sold commercially (Patel 2012). In vitro studies of *P. cattleianum* revealed that this plant can be considered a good source of bioactive compounds with antioxidant, antihyperglycemic, and antidyslipidemic effects (de Souza et al. 2018). Moreover, phytochemical investigations of leaf extracts resulted in the isolation of two new meroterpenoid phloroglucinols (cattleianone and cattleianal) with selective cytotoxic activity against the human cancer cell lines MDA‐MB231, HepG‐2, HT‐29, and MCF7 (Mahrous et al. 2021). In parallel, leaf extract presented satisfactory antimicrobial effects against important pathogens, especially against *Enterococcus faecalis* and *Actinomyces israelii* , associated with persistent endodontic infections (Massunari et al. 2017). In addition, other studies revealed that fruits have neurochemical, metabolic, and behavioral benefits in an animal model of metabolic syndrome (Oliveira et al. 2018) and antibacterial activity against *Salmonella enteritidis* and *Staphylococcus aureus* (Lisboa et al. 2011; Mccook‐Russell et al. 2012).

Therefore, the vast array of biological properties of *P. cattleianum* extracts is well established, but the mechanism of action is unrevealed. Indeed, transient receptor potential (TRP) channels are engaged in many signaling pathways through modulation of ion exchange (Ca^2+^, Mg^2+^) across the plasma membrane. In this sense, in the past few years, the knowledge about the range of potential clinical indications of the molecules targeting TRP channels has grown. Recent reports suggest the possible use of such compounds in neurological, psychiatric, respiratory diseases, diabetes, and cancer (Koivisto et al. 2022). Furthermore, TRP channels are molecular targets of many natural products, especially those with anti‐inflammatory and analgesic activities. A significant group of natural compounds acting through TRP ion channels is phenolics (Meotti et al. 2014). Thus, TRPA1 and TRPV1 channels were chosen as potential biological targets for the mode of action of *P. cattleianum* extracts that have a rich phenolic composition.

Remarkably, there is a limited information regarding the phytochemical profile of *P. cattleianum* . The few available studies have mostly addressed the plant metabolites characterization by LC‐DAD‐ESI‐MS/MS and LC–MS/MS in red and yellow fruit genotypes from Brazilian cultivars of *P. cattleianum* , in addition to GC–MS analysis of the Brazilian leaf essential oil (de Souza et al. 2021; Mallmann et al. 2020; Schulz et al. 2020; Scur et al. 2016; Vasconcelos et al. 2019). Additionally, Campos et al. (2021) studied the volatile oil composition in *P. cattleianum* leaves from Polynesia, Hawaii, and Cuba.

Previous studies have investigated essential oils and some phenolic metabolites in the chloroform: methanol (80:20) extract of *P. cattleianum* leaves collected from Egypt (Saber et al. 2018; Soliman et al. 2016). Therefore, in order to extend our findings, the present study focused on unraveling the variation of chemical profiles of the more polar alcoholic extracts of *P. cattleianum* fruits and leaves by an untargeted approach via ultra‐high pressure liquid chromatography coupled with quadrupole‐time‐of‐flight mass spectrometry (UHPLC‐QTOF‐HRMS). This technique has been useful as a fingerprinting approach to trace medicinal plants from different geographical regions and for evaluating the metabolic variances in different plant parts (Paula et al. 2020; Zhang, Rocchetti, et al. 2021; Zhang, Saber, et al. 2021). Therefore, the importance of this work lies in acquiring information on the different types of phytochemicals in the two plant organs so that it validates a basis for pharmacological and nutraceutical exploitation. Accordingly, a complete phytochemical profile of fruit and leaf extracts of a *P. cattleianum* plant cultivated in Egypt is developed. Furthermore, the assessment of the biological potential of *P. cattleianum* extracts against TRP receptors was investigated, integrating both in vitro and *in silico* studies.

## 2 Material and Methods

### 2.1 Plant Material and Extraction

The plant material of *Psidium cattleianum* Sabine was collected in May 2020 from the Experimental Station of Medicinal Plants, Faculty of Pharmacy, Cairo University. Plants are cultivated, harvested, and collected at the experimental station in accordance with national guidelines of the Agriculture Research Center, Giza, Egypt, and solely for research and educational purposes. The Samples were kindly identified by Dr. Mohamed El‐Gebaly (Senior Botanist). A voucher specimen (PC‐24‐5‐2020) was kept at the herbarium of the Department of Pharmacognosy, Faculty of Pharmacy, Cairo University. Leaves and fruits were collected from 3 different 5‐year‐old trees. Then, leaves were dried in the shade, powdered, and stored in airtight containers. However, the fruit pericarps were frozen in liquid nitrogen and kept at −80°C until further analysis.

The powdered plant material from fruits and leaves (100 g each) was subjected to ultrasonic‐assisted extraction using 1 L methanol (Sigma‐Aldrich, analytical grade) at 50°C for 30 min as the extracting solvent. Extraction of plant material was done in triplicate.

### 2.2 Bioactive Compound Profiling Using UHPLC‐QTOF Mass Spectrometry

The phytochemical profile of *P. cattleianum* extracts was assessed using an untargeted metabolomics approach. To this aim, methanolic extracts were analyzed by ultra‐high performance liquid chromatography coupled to quadrupole time‐of‐flight mass spectrometry (UHPLC‐QTOF‐MS) as previously reported (Zhang, Rocchetti, et al. 2021). Briefly, reverse phase chromatography with a 34‐min binary gradient elution (acetonitrile and water), together with high‐resolution MS SCAN (100–1200 *m/z*), was used. The injection volume was 6 μL and compounds annotation was achieved according to the whole isotopic pattern of deconvoluted features (monoisotopic mass, isotope spacing and ratio) following mass and retention time alignment. Therefore, a level 2 of confidence in annotation was achieved, with reference to COSMOS standards in metabolomics (Salek et al. 2013). The extraction procedure, instrumental conditions for untargeted analysis, and compound annotation procedure are reported in detail as Data S1.

### 2.3 Chemometrics Analysis

The data derived from UHPLC‐QTOF‐MS were elaborated using the software Mass Profiler Professional 12.6 (Agilent Technologies). The raw data were log2 transformed, 75th percentile normalized, and baselined against the median of each compound. Sample profiles were investigated using both unsupervised and supervised multivariate statistics using MetaboAnalyst 6.0 (https://www.metaboanalyst.ca), where hierarchical cluster analysis (HCA; unsupervised) was used to highlight patterns across the different treatments, and orthogonal projection to latent structures discriminant analysis (OPLS‐DA; supervised) was carried out as a supervised model to select Variable Importance in Projection (VIP > 1.2) markers for leaves and fruits of *P. cattleianum* .

### 2.4 TRP Assay

*P. cattleianum* leaves and fruit extracts were tested in human embryonic kidney (HEK‐293) cells stably transfected with rat TRPA1 or human TRPV1 to evaluate the intracellular Ca^2+^ concentration ([Ca^2+^]_i_). Untransfected cells were used as the internal control. Briefly, on the day of the experiment, control and transfected cells were loaded with methyl ester Fluo4‐AM (4 μM in DMSO containing 0.02% Pluronic F‐127, Invitrogen, Carlsbad, CA, USA) in EMEM without fetal bovine serum (FBS) for 1 h in the dark at room temperature. After incubation, the cells were rinsed and resuspended in Tyrode's solution (145 mM NaCl, 2.5 mM KCl, 1.5 mM CaCl_2_, 1.2 mM MgCl_2_, 10 mM D‐glucose, and 10 mM HEPES, pH 7.4). Subsequently, the cells were collected and transferred to a quartz cuvette of a spectrofluorimeter (Perkin‐Elmer LS50B; λEX = 488 nm, λEM = 516 nm). Fluorescence signal intensity was measured before and after the stimulation with crescent concentrations of *P. cattleianum* extracts. The efficacy of each extract is reported as normalized to the response of 4 μM ionomycin (Cayman, USA). The signal intensity obtained in non‐transfected HEK‐293 cells was used as the baseline and subtracted as background. The effects of *P. cattleianum* extracts on TRPA1 are reported as a percentage of the value of 100 μM allyl isothiocyanate (AITC). The potency is expressed as the concentration of extracts exerting a half‐maximal agonist effect (i.e., half‐maximal increases in [Ca^2+^]_i_ (EC_50_)). The TRPA1 or TRPV1 channels desensitization (reported as antagonism) was calculated by measuring the effect of *P. cattleianum* extracts preincubated into the cells 5 min before the stimulation with AITC 100 μM (TRPA1 agonist) or capsaicin 0.1 μM (TRPV1 agonist). IC_50_ is expressed as the concentration of *P. cattleianum* extracts exerting a half‐maximal inhibition on the agonist response. Dose–response curve fitting (sigmoidal dose–response variable slope) and parameter estimation were analyzed using Graph‐Pad Prism8 (GraphPad Software Inc., San Diego, CA, USA).

### 2.5 Molecular Docking

Molecular docking was performed using the Molecular Operating Environment software (MOE—v.2019.01) (Molecular Operating Environment 2019). Structures of the compounds analyzed in this study were obtained from the NIH (National Institution of Health)‐PubChem Website (https://pubchem.ncbi.nlm.nih.gov/) by retrieving their isomeric SMILES representations and preparing a database therefrom. Conformational search was performed on all the analyzed compounds and the lowest energy conformer was employed for the docking protocol. TRPA1 electron microscopy‐obtained crystal structure (PDB ID: 6X2J) was retrieved from the protein databank and utilized for the docking study. Upon searching for TRPA1 crystal structures in the RCSB database, our PDB structure was judged as the best candidate for our study for the following reasons. It is a homosapiens structure with acceptable resolution among other structures (3 Å). Further, It is the only structure crystallized with an agonist thus having a suitable active site conformation for the docking study.

Before docking, protein preparation was performed by adding hydrogens and removing two chains out of the four identical chains of the homotetrameric protein structure to focus on one agonist site at the interface of two adjacent chains. The forcefield was set to Amber10, and the compounds were docked at the agonist site of the crystal structure of TRPA1 (PDB ID: 6X2J) keeping all parameters as default to assess accommodation of the ligand binding. Docking validation was carried out by executing the docking on the co‐crystallized ligand 5‐amino‐1‐[(4‐bromo‐2‐fluorophenyl)methyl]‐N‐(2,5‐dimethoxyphenyl)‐1H‐1,2,3‐triazole‐4‐carboxamide (PDB ID: ULJ) applying the same docking protocol. All interactions were analyzed through the Protein Ligand Interaction Profiler Webtool (https://plip‐tool.biotec.tu‐dresden.de/plip‐web/plip/index) and their results were visualized on Pymol software (The PyMOL Molecular Graphics System 2002).

### 2.6 Molecular Dynamics Simulation

In order to investigate the stability of the binding and interaction of the proposed candidates with the TRPA1 ion channel (PDB ID: 6X2J), Molecular Dynamics (MD) simulation was conducted, where theaflavin 3,3'‐*O*‐digallate was chosen due to its promising docking results. The simulation was executed for 100 ns by the aid of GROMACS‐2022, where the CHARMM36 force field (Huang and MacKerell Jr 2013) was employed during the protein topology and The Official CHARMM Force Field Server (CGenFF) was employed for the ligand topology preparation (Brooks et al. 2009; Lee et al. 2023). For the environment preparation, a triclinic unit cell was implemented, followed by solvation with water molecules and then neutralization by the addition of sodium and chloride ions. To ensure an optimized, clashes‐eliminated system, energy minimization was conducted employing the steepest descent algorithm, with an energy cut‐off of 10 kJ/mol, and a maximized limit of 50,000 steps was applied. Subsequently, the system's Berendsen thermostat and the Leap‐frog integrator were adjusted in two steps, starting with the volume fixation with NVT and then pressure fixation through NPT. The final step was the MD production, which was anticipated to last for 100,000 ps with a time frame of 2 ps per step.

## 3 Results and Discussion

### 3.1 Phytochemical Profiling of *P. cattleianum* by UHPLC‐QTOF Mass Spectrometry

**FIGURE 1:**
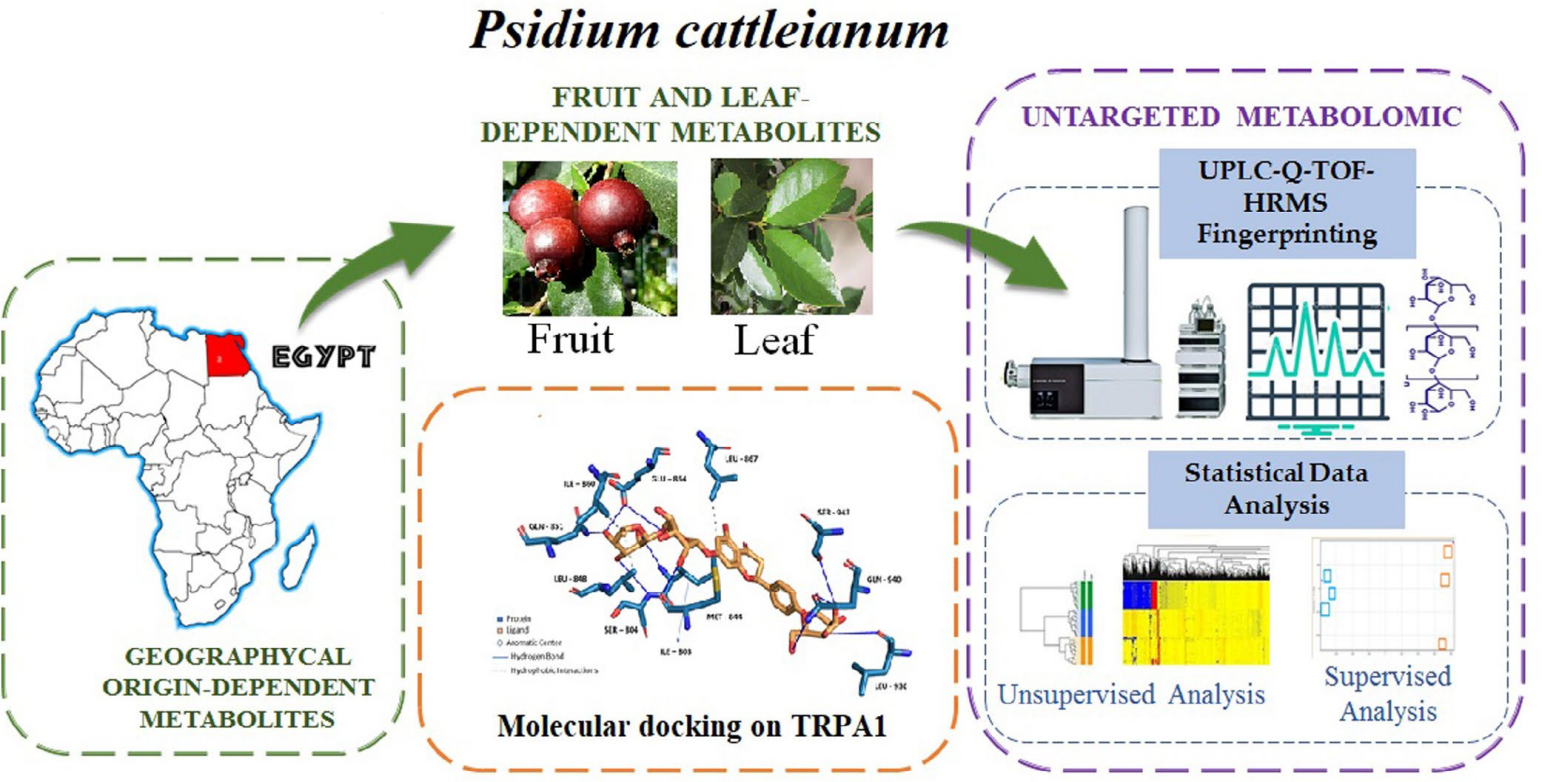
Schematic representation of untargeted metabolomic analysis of *Psidium cattleianum* extracts of leaves and fruits growing in Egypt.

A comprehensive study focused on the phenolic profiling of leaves and fruits of *P. cattleianum* was performed using an untargeted metabolomic approach (Figure 1). This method allowed the annotation of 465 phytochemicals, predominantly composed of the flavonoid group, including anthocyanins, flavonols, flavanols, flavones, flavanones, isoflavonoids, chalcones, dihydrochalcones, and dihydroflavonols (Table S1). The study also revealed the presence of phenolic acids from the hydroxycinnamic group, low‐molecular‐weight (LMW) phenolics such as tyrosols, alkylphenols, and hydroxycoumarins, along with stilbenes and lignans. Notably, this profile is similar to that obtained from *P. cattleianum* leaves ethanolic:water extracts of a Brazilian yellow fruit variety, as profiled using a UPLC‐QTOF‐MS. Where the identified several compounds belonged to flavonoids, phenolic acids (hydroxycinnamic and hydroxybenzoic), tannins (hydrolyzable and condensed), carbohydrates, and phenol lipids (Paula et al. 2020). Interestingly, the phenolic profile of the Egyptian red cultivar appears to share similarities with the Brazilian yellow fruits, particularly in its enrichment with hydroxybenzoic and hydroxycinnamic acids, as well as procyanidins (Paula et al. 2020). Furthermore, the investigated *P. cattleianum* extracts were found to contain tyrosol phenolics (tyrosol, hydroxytyrosol and its glycoside), alkylphenols (5‐heneicosylresorcinol, 5‐pentacosenylresorcinol), and hydroxycoumarins (esculetin). A tyrosol ester derivative named araçain was recently characterized from the ethyl acetate extract of *P. guineense* leaves. Moreover, in both leaf and fruit profiles, flavonoids emerged as the most predominant group based on the number of identified compounds. The presence of morin, quercetin, myricetin, and their glycosylated forms aligns with previous reports (Ho et al. 2012; Saber et al. 2018). Notably, isorhamnetin and its derivatives are detected in *P. cattleianum* extracts for the first time.

**FIGURE 2:**
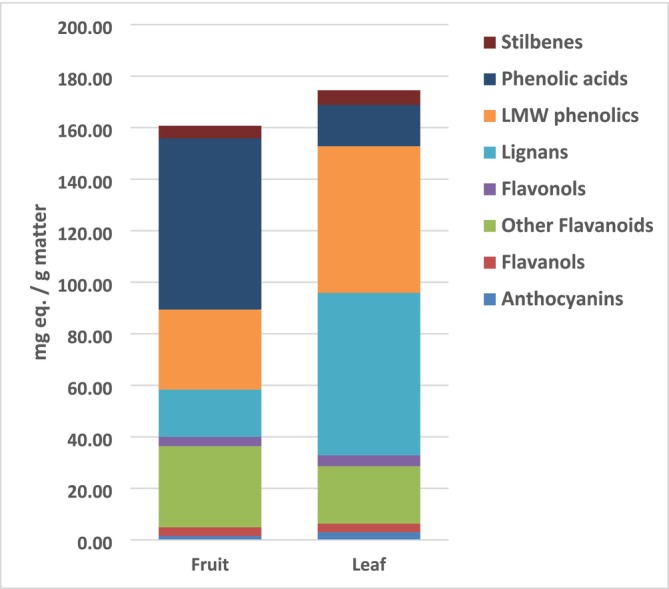
The semi‐quantification profile of the different phenolic classes of the extracts from the fruit and leaves of *P. cattleianum* . Results are expressed as mg Equivalent (Eq.)/g considering the mean value of *n* = 3 experiments. LMW, lower‐molecular‐weight phenolics (i.e., tyrosol derivatives).

The semi‐quantification per class of the different phytochemicals was performed using representative standard compounds, and the results are depicted in Figure 2 and detailed values are provided in Table 1. the total phenolic content (TPC) was 160.71 mg Eq/g in fruits and 174.51 mg Eq/g in leaves, showing distinct chemical profiles. In fruits, phenolic acids were the predominant group (66.47 mg Ferulic acid Eq/g), followed by LMW phenolics (31.06 mg Tyrosol Eq/g) and the combined flavones, flavanones, and isoflavonoids categorized as “other flavonoids” (31.5 mg Luteolin Eq/g). The content of lignans was also quite significant at levels of 18.3 mg sesamin Eq/g. However, anthocyanins, flavonols, flavanols, and stilbenes were found in smaller amounts, ranging from 1.54 to 4.82 mg Eq/g.

**TABLE 1:** Semi‐quantification of different phenolic classes in *P. cattleianum* extracts.

	Anthocyanins	Flavanols	Other flavonoids	Flavonols	Lignans	LWM phenolics	Phenolic acids	Stilbenes
Fruit	1.54 ± 0.23	3.35 ± 0.17	31.51 ± 1.02^a^	3.63 ± 0.84	18.34 ± 1.60^b^	31.06 ± 1.42^b^	66.47 ± 3.74^a^	4.82 ± 0.32
Leaf	3.00 ± 0.41^a^	3.32 ± 1.02	22.28 ± 0.19^b^	4.27 ± 0.20	62.98 ± 4.16^a^	56.95 ± 5.10^a^	15.99 ± 1.24^b^	5.72 ± 1.96

*Note:* Values are reported as mean ± SD calculated from three parallel measurements (*n* = 3). Different letters within each column indicate t‐test (*p*‐value < 0.05).

Abbreviation: ns, nonsignificant.

Conversely, in leaves, lignans were the most abundant class of compounds (62.98 mg sesamin Eq/g), followed by the LMW phenolics (56.95 mg Tyrosol Eq/g) and the combined flavones, flavanones, and isoflavonoids (22.28 mg Luteolin Eq/g). Phenolic acids were found in lower amounts (15.99 mg Ferulic acid Eq/g), and, like fruits, anthocyanins, flavonols, flavanols, and stilbenes were present in smaller quantities, ranging from 3.0 to 5.72 mg Eq/g.

The results revealed that the main differences in the phytochemical profile between fruits and leaves are found in the phenolic acid and lignan content. Notably, the phenolic acids, mostly exemplified by hydroxycinnamic and hydroxybenzoic acids, were represented in fruits 4 times more than in leaves. Specifically, within the phenolic acids class, the most abundant compounds characterizing this category in both leaves and fruits of *P. cattleianum* were identified as cinnamic acid, *p*‐coumaric acid ethyl ester, ferulate steroid esters, 1‐sinapoyl‐2‐feruloylgentiobiose, 1,2,2′‐triferuloylgentiobiose, 2,5‐di‐S‐glutathionyl caftaric acid, 1‐sinapoyl‐2,2′‐diferuloylgentiobiose, hydroxybenzoic acid, galloyl glucose, and ellagic acid derivatives (ellagic acid acetyl‐arabinoside, ellagic acid glucoside and ellagic acid arabinoside). Importantly, this study revealed a more extensive and diverse range of phenolic compounds than previous investigations of yellow guava ( *P. cattleianum* Sabine) fruits, employing targeted analytical approaches (LC–MS/MS), which identified 23 compounds at different ripening stages (Schulz et al. 2020). Among them, catechin, isoquercitrin, quercetin, gallic acid, and syringic acid showed significant concentrations, with catechin as the predominant compound constituting up to 0.13 mg/g DM. Furthermore, this study identified a broader spectrum of compounds, with quercetin and several epicatechin derivatives dominating the strawberry guava ( *P. cattleianum* ) phytochemical profile. Interestingly, epicatechin, ellagic acid, gallic acid, and quercetin were previously described as being associated with anti‐inflammatory, antiproliferative, and antimicrobial effects, as well as chelating properties and the maintenance of the endogenous antioxidant defense system (dos Santos Pereira et al. 2018).

On the other hand, lignans (as represented by secoisolariciresinol‐sesquilignan) and the LMW phenolics like 5‐heneicosylresorcinol, 5‐pentacosenylresorcinol, hydroxytyrosol, esculetin, and pyrogallol were found in higher abundance (about triple and double, respectively) in the leaves compared to the fruits extract of *P. cattleianum* . In a previous untargeted profile analysis of *P. cattleianum* leaf extracts, various compounds were quantified, including vanillic, syringic, and gallic acid, catechin, myricetin, quercetin, kaempferol, and luteolin (Paula et al. 2020). This chemical profile aligns with the present study in which quercetin and its derivatives, myricetin, kaempferol, and luteolin were found in the *P. cattleianum* leaf extract.

The current findings reported molecules with high antioxidant potential belonging to the tyrosol phenolic group (hydroxytyrosol), not previously described in *P. cattleianum* . Only a limited number of research articles report the presence of tyrosol derivatives in leaf extracts of *Psidium* species, including *P. guineense* (Karoline et al. 2019). Hydroxytyrosol is a common phenolic found in *Olea europaea* L. leaf extracts (Galanakis et al. 2018; Otero et al. 2021), known for its high antioxidant capacity and significant biological properties (Cea Pavez et al. 2019; Vilaplana‐Pérez et al. 2014). To the best of our knowledge, the presence of hydroxytyrosol and its glycoside in *P. cattleianum* leaf extracts is not recorded. As previously suggested in the literature, it could contribute to its high antioxidant properties (Mccook‐Russell et al. 2012; de Souza et al. 2021).

### 3.2 Multivariate Data Analysis Following Fruits and Leaves Phytochemical Profiling

**FIGURE 3:**
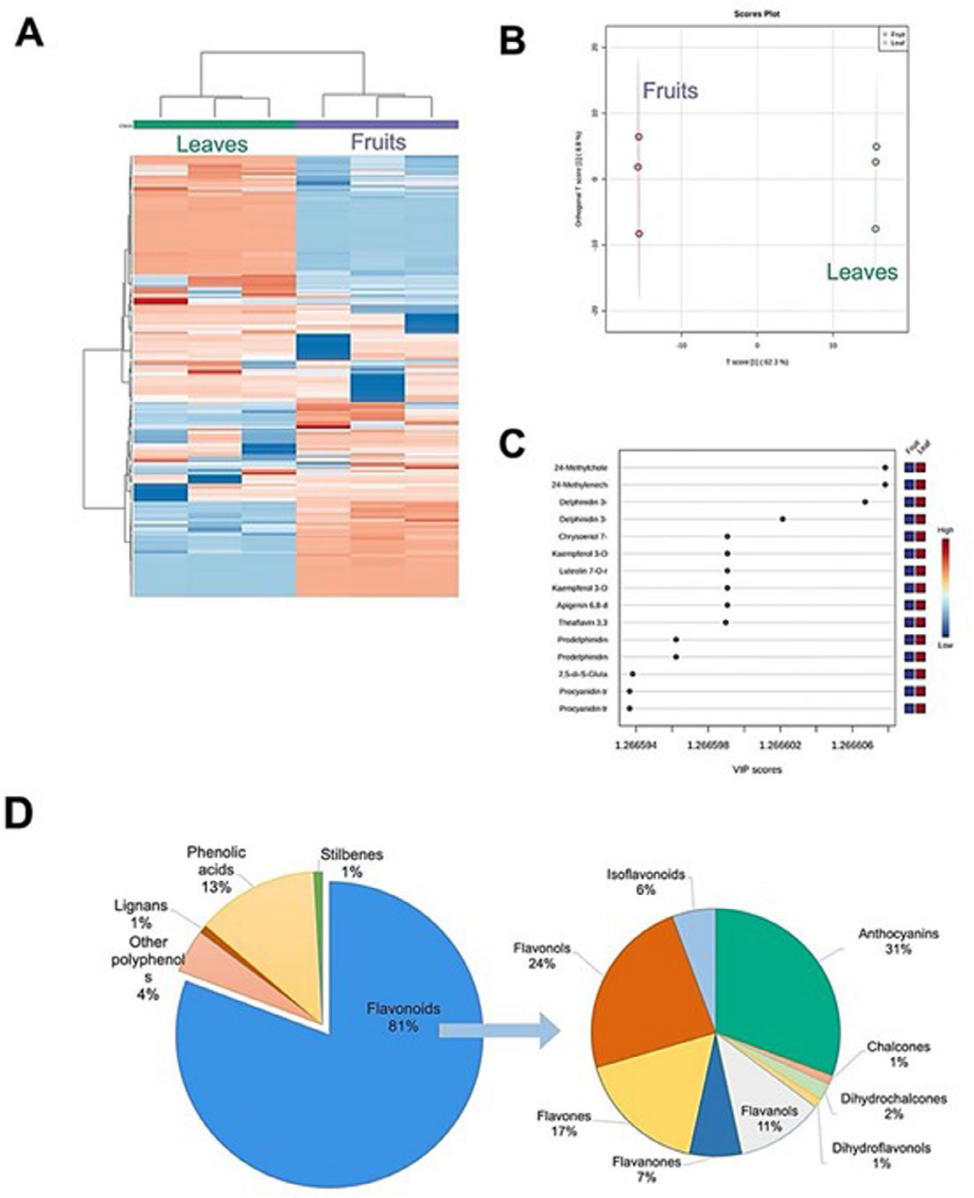
(A) Unsupervised hierarchical cluster analysis (HCA) built according to the fold‐change heatmap based on flavonoids and phenolics in fruit and leaf extracts of *P. cattleianum*. The cluster was built using Log 10 median normalized values (similarity: Squared Euclidean; linkage rule: Ward). The heat‐map color range in each column represents the maximum (red) and minimum (blue) fold‐change values. (B) Orthogonal Partial Least Square Data Analysis model. (C) Variable Importance on Projection plot, considering vector 1. (D) Pie chart of different phenolic classes of VIP markers obtained from OPLS‐DA model.

Supervised and unsupervised multivariate statistical analyses were used as a data reduction approach. The unsupervised hierarchical cluster analysis (HCA) resulting from the heatmap (produced from the fold‐change of each annotated compound in fruit and leaf extracts) is shown in Figure 3A. This methodology highlighted the similarities/dissimilarities across the different plant parts, showing two main clusters. Fruits demonstrated a very different profile compared to the leaves. The supervised Orthogonal Partial Least Square Data Analysis (OPLS‐DA; Figure 3B) was performed to identify the compounds that better discriminate between fruit and leaf extracts of *P. cattleianum* . In this sense, the variable importance of projection (VIP) markers was determined to better explain the phenolic differences between the two organs (Table 2). Table S2 reported the VIP markers that are statistically different and have high LogFC variation, determined by pairwise comparison among leaf and fruit extracts.

**TABLE 2:** VIP markers of the OPLS‐DA model completed with volcano analysis obtained by pairwise comparison among leaf and fruit extracts of *P. cattleianum* .

Class	Subclass	Discriminant compound	VIP comp1	*p* (corr)	Log FC [leaf] versus [fruit]
Flavonoids	Anthocyanins	Delphinidin 3*‐O‐*(6″‐acetyl‐galactoside)	1.267	0.000	20.23
		Delphinidin 3*‐O‐*galactoside	1.267	0.000	10.29
		Peonidin 3*‐O‐*galactoside	1.267	0.000	21.32
		Peonidin 3*‐O‐*(6″‐acetyl‐galactoside)	1.267	0.000	19.86
		Petunidin 3,5*‐O‐*diglucoside	1.267	0.000	19.40
		Cyanidin 3*‐O‐*(6″‐malonyl‐3″‐glucosyl‐glucoside)	1.267	0.000	19.48
		Pinotin A/Petunidin 3*‐O‐*(6″‐p‐coumaroyl‐glucoside)	1.267	0.000	19.48
		Delphinidin 3*‐O‐*feruloyl‐glucoside	1.267	0.000	19.48
		Delphinidin 3,5*‐O‐*diglucoside/Delphinidin 3*‐O‐*glucosyl‐glucoside	1.267	0.000	19.49
		Malvidin 3*‐O‐*(6″‐p‐coumaroyl‐glucoside)	1.267	0.000	18.65
		Cyanidin 3*‐O‐*(6″‐dioxalyl‐glucoside)	1.267	0.000	18.57
		Malvidin 3*‐O‐*(6″‐acetyl‐galactoside)	1.267	0.000	19.61
		Pelargonidin 3*‐O‐*glucosyl‐rutinoside	1.266	0.000	20.60
		Cyanidin 3*‐O‐*sophoroside/Cyanidin 3,5*‐O‐*diglucoside	1.266	0.000	−10.29
		Petunidin 3*‐O‐*galactoside	1.266	0.000	19.69
		Delphinidin 3*‐O‐*(6″‐acetyl‐glucoside)	1.266	0.000	17.42
		Petunidin 3*‐O‐*rhamnoside	1.265	0.000	−9.31
		Pelargonidin 3*‐O‐*sambubioside	1.265	0.000	−18.59
		Malvidin 3*‐O‐*glucoside	1.265	0.000	−9.82
		Cyanidin 3*‐O‐*(6″‐p‐coumaroyl‐glucoside)	1.264	0.000	−17.99
		Cyanidin 3*‐O‐*xyloside/Cyanidin 3*‐O‐*arabinoside	1.264	0.000	−9.62
		Petunidin 3*‐O‐*(6″‐acetyl‐galactoside)	1.264	0.000	−18.55
		Peonidin	1.262	0.000	−22.03
		Malvidin 3*‐O‐*(6″‐acetyl‐glucoside)	1.258	0.000	−19.43
		Cyanidin 3*‐O‐*glucosyl‐rutinoside	1.254	0.000	−10.16
		Malvidin 3*‐O‐*arabinoside	1.247	0.000	−20.14
		Peonidin 3*‐O‐*glucoside	1.247	0.000	−21.44
	Chalcones	Butein	1.255	0.000	−20.11
	Dihydrochalcones	3‐Hydroxyphloretin 2'*‐O‐*xylosyl‐glucoside	1.267	0.000	−11.00
	Dihydroflavonols	Phloretin 2'*‐O‐*xylosyl‐glucoside	1.266	0.000	−19.36
		Dihydroquercetin	1.258	0.000	20.65
	Flavanols	Theaflavin 3,3'*‐O‐*digallate	1.267	0.000	17.97
		Prodelphinidin trimer C‐GC‐C	1.267	0.000	16.83
		Procyanidin trimer C1	1.267	0.000	−17.87
		Procyanidin trimer C2	1.267	0.000	−9.20
		Procyanidin trimer EEC	1.267	0.000	−8.37
		Prodelphinidin trimer GC–GC‐C	1.267	0.000	−10.47
		(−)‐Epicatechin‐(2a‐7) (4a‐8)‐epicatechin 3*‐O‐*galactoside	1.267	0.000	18.58
		Theaflavin 3*‐O‐*gallate	1.267	0.000	−17.10
		Spinacetin 3*‐O‐*glucosyl‐(1–6)‐[apiosyl(1–2)]‐glucoside	1.267	0.000	22.77
		Cannflavin A	1.266	0.000	−21.35
	Flavanones	Naringin 4'*‐O‐*glucoside	1.267	0.000	−19.97
		Naringin 6'‐malonate	1.267	0.000	19.70
		Narirutin 4'*‐O‐*glucoside	1.267	0.000	−20.56
		Narirutin/Naringin	1.266	0.000	−21.15
		6‐Geranylnaringenin	1.265	0.000	19.06
		Naringenin	1.255	0.000	18.87
	Flavones	Chrysoeriol 7*‐O‐*apiosyl‐glucoside/Luteolin 7*‐O‐*rutinoside/Apigenin 6,8‐di‐C‐glucoside	1.267	0.014	1.35
		Apigenin 7*‐O‐*diglucuronide	1.267	0.000	17.62
		Apigenin 6,8‐C‐arabinoside‐C‐glucoside	1.267	0.001	3.11
		Luteolin 7*‐O‐*malonyl‐glucoside	1.266	0.000	3.91
		Rhoifolin/Isorhoifolin	1.266	0.000	9.89
		Luteolin 7*‐O‐*diglucuronide	1.265	0.000	−3.65
		6‐Hydroxyluteolin	1.265	0.004	1.96
		Luteolin 7*‐O‐*(2‐apiosyl‐6‐malonyl)‐glucoside	1.264	0.004	1.48
		Hispidulin	1.259	0.001	−3.07
		Nobiletin	1.258	0.000	−19.03
		Chrysoeriol 7*‐O‐*glucoside	1.257	0.007	1.63
		7,4'‐Dihydroxyflavone	1.254	0.000	−3.49
		Chrysin	1.254	0.000	−3.49
		Apigenin 7*‐O‐*(6″‐malonyl‐apiosyl‐glucoside)	1.252	0.000	−3.49
		Neodiosmin/Diosmin	1.238	0.000	9.06
	Flavonols	Quercetin 3*‐O‐*xylosyl‐glucuronide	1.267	0.000	9.54
		5,3′,4′‐Trihydroxy‐3‐methoxy‐6:7‐methylenedioxyflavone 4'*‐O‐*glucuronide	1.267	0.000	−9.42
		Kaempferol 3,7,4'*‐O‐*triglucoside	1.267	0.000	−2.72
		Quercetin 3*‐O‐*rhamnosyl‐rhamnosyl‐glucoside	1.267	0.004	2.21
		Isorhamnetin 4'*‐O‐*glucoside	1.267	0.005	1.93
		Quercetin 3*‐O‐*xylosyl‐rutinoside	1.266	0.001	3.78
		Myricetin 3*‐O‐*galactoside	1.266	0.001	2.82
		Quercetin 3*‐O‐*xyloside	1.266	0.001	2.82
		Myricetin 3*‐O‐*arabinoside	1.265	0.001	2.82
		Methylgalangin	1.265	0.004	4.29
		Isorhamnetin 3*‐O‐*glucuronide	1.265	0.000	18.07
		Kaempferol 7*‐O‐*glucoside	1.265	0.010	1.37
		Morin	1.265	0.001	20.44
		Quercetin	1.265	0.010	1.83
		Kaempferol 3*‐O‐*sophoroside/Kaempferol 3,7*‐O‐*diglucoside	1.264	0.002	−3.56
		Kaempferol 3*‐O‐*rhamnoside	1.263	0.010	1.75
		Quercetin 3*‐O‐*glucosyl‐xyloside	1.262	0.011	−1.78
		Isorhamnetin 3*‐O‐*glucoside	1.261	0.002	3.83
		Isorhamnetin 7*‐O‐*rhamnoside	1.261	0.008	−1.72
		3‐Methoxysinensetin	1.258	0.006	2.41
		Isorhamnetin 3*‐O‐*rutinoside	1.257	0.006	−2.76
	Isoflavonoids	6″‐*O*‐Malonyldaidzin	1.267	0.019	−3.59
		6″‐*O*‐Acetylglycitin	1.265	0.019	−3.59
		Biochanin A	1.265	0.025	−1.66
		Glycitein	1.265	0.048	−2.35
		Daidzein	1.254	0.029	−2.21
Lignans	Lignans	Secoisolariciresinol‐sesquilignan	1.008	0.029	−2.21
Other polyphenols	Alkylphenols	5‐Heneicosylresorcinol	1.267	0.000	19.92
		5‐Pentacosenylresorcinol	1.263	0.000	20.90
	Hydroxycoumarins	Esculetin	1.252	0.019	−3.59
	Other polyphenols	Pyrogallol	1.257	0.029	−1.37
	Tyrosols	Hydroxytyrosol	1.221	0.000	3.61
Phenolic acids	Hydroxybenzoic acids	Ellagic acid acetyl‐arabinoside	1.267	0.010	−2.68
		Galloyl glucose	1.267	0.010	−2.68
		2‐Hydroxybenzoic acid/3‐Hydroxybenzoic acid/4‐Hydroxybenzoic acid	1.265	0.025	2.43
		Ellagic acid arabinoside	1.262	0.011	3.99
		Ellagic acid glucoside	1.258	0.010	4.75
	Hydroxycinnamic acids	24‐Methylcholesterol ferulate/24‐Methylenecholestanol ferulate	1.267	0.010	4.75
		2,5‐di‐S‐Glutathionyl caftaric acid	1.267	0.010	−5.06
		Stigmastanol ferulate	1.267	0.012	2.19
		1‐Sinapoyl‐2,2′‐diferuloylgentiobiose	1.267	0.004	−1.77
		1,2,2′‐Triferuloylgentiobiose	1.267	0.003	−2.67
		Cinnamic acid	1.265	0.003	−2.67
		24‐Methyllathosterol ferulate	1.265	0.028	−3.31
		24‐Methylcholestanol ferulate	1.264	0.028	−3.31
		p‐Coumaric acid ethyl ester	1.263	0.015	−2.51
Stilbenes	Stilbenes	Piceatannol	1.267	0.043	−1.62

Abbreviation: FC, fold change.

As reported in Figure 3D, the most discriminant metabolites of *P. cattleianum* fruits and leaves were flavonoids (81%), characterized by anthocyanins (31%), flavonols (24%), flavones (17%), and other flavonoids contributing to a lesser extent. Furthermore, phenolic acids were reported to have great discriminant potential, followed by other polyphenols, lignans, and stilbenes. Notably, the hydroxycinnamic acid compounds, particularly 24‐methylcholesterol ferulate/24‐methylenecholestanol ferulate, displayed the highest discriminant potential (Figure 3C). Within the flavonoid classes, anthocyanins such as delphinidin 3‐*O*‐(6″‐acetyl‐galactoside) and delphinidin 3‐*O*‐galactoside, as well as flavones including chrysoeriol 7‐*O*‐apiosyl‐glucoside/luteolin 7‐*O*‐rutinoside/apigenin 6,8‐di‐*C*‐glucoside, exhibited significant discriminant potential, found to be highly abundant in leaves (Figure 3C). Finally, other flavonoids like prodelphinidin trimer C‐GC‐C and prodelphinidin trimer GC‐GC‐C were identified as important VIP markers.

### 3.3 Evaluation of the Effect of the Extracts on Transient Receptor Potential Channels TRPV1 and TRPA1

To shed light on the potential action of *P. cattleianum* leaves and fruits in eukaryotic cells, the activity of their extracts was explored on TRP channels. These channels represent a large family of cations (mostly Ca^2+^, Na^+^ and Mg^2+^) permeable channel‐receptors best known for their ability to be gated by endogenous molecules, numerous chemicals, and physical stimuli (Zheng 2013). In particular, the *P. cattleianum* leaf and fruit extracts were tested on Transient receptor potential vanilloid 1 (TRPV1) and Transient receptor potential ankyrin 1 (TRPA1), which are two of the most ubiquitously expressed members in their respective subfamilies within the body. Therefore, the effects of *P. cattleianum* extracts on the intracellular Ca^2+^ concentration ([Ca^2+^]_i_) were measured in human embryonic kidney (HEK) 293 cells stably transfected with rat TRPA1 or human TRPV1 channels. In the TRPA1 assay, changes in the [Ca^2+^]_i_ induced by *P. cattleianum* extracts were normalized (%) to that of AITC, a selective and potent TRPA1 agonist. In addition, TRPA1 desensitization, which is a known functional property of all these channel‐receptors that causes their inactivation following prolonged exposure to agonists (Iannotti et al. 2014), was evaluated by preincubating the cells with *P. cattleianum* leaf and fruit extracts for 5 min before stimulation with AITC. Likewise, the effects on TRPV1 desensitization were evaluated using capsaicin, a selective agonist.

**FIGURE 4:**
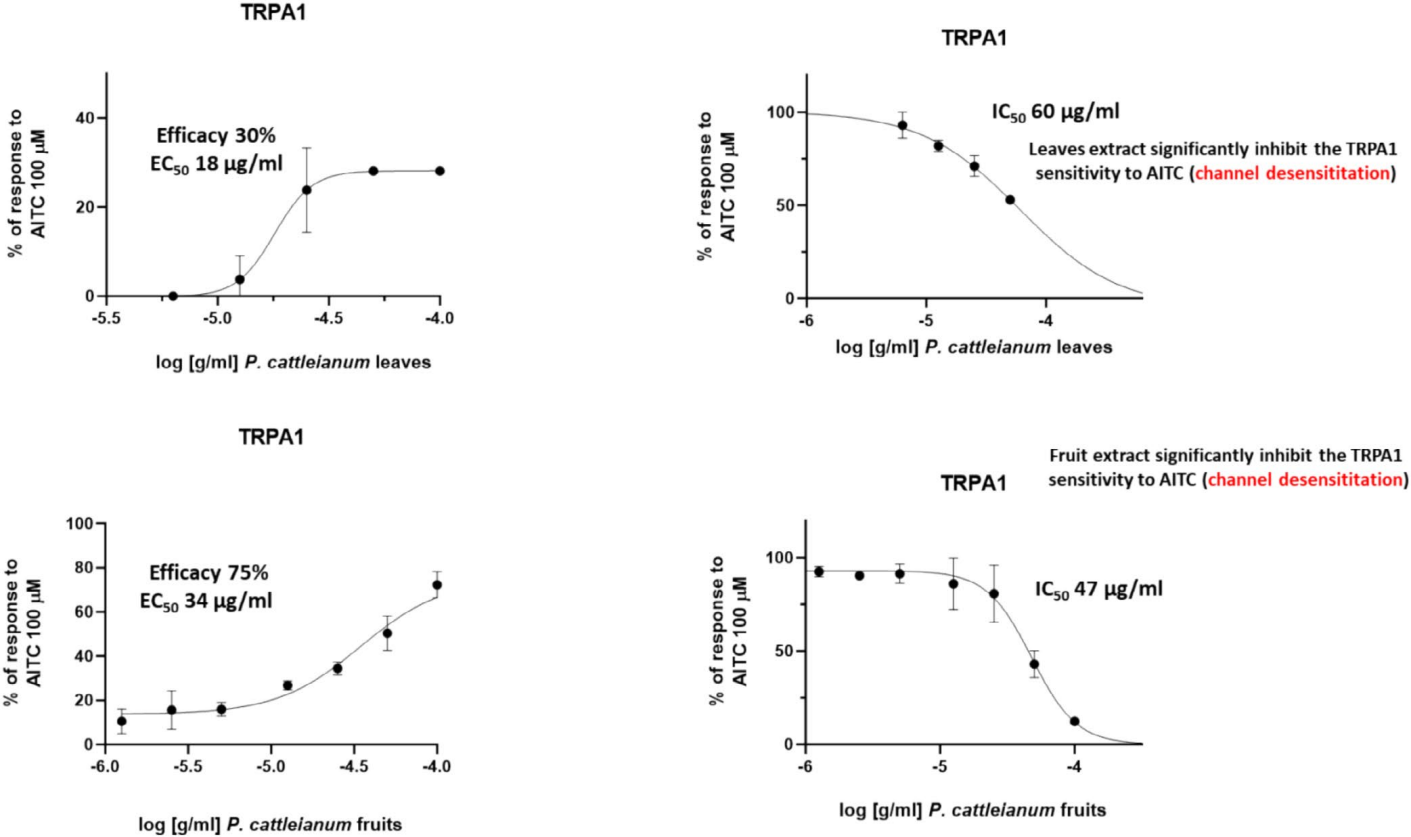
Effect of *P. cattleianum* leaf and fruit extracts on TRPA1 channels. Concentration‐response curves showing the activity of *P. cattleianum* leaves and fruits on TRPA1 channels calculated log(concentration) versus response—variable slope. AITC (100 μM) were used to calculate the % of *P. cattleianum* response or induced desensitization.

It was found that *P. cattleianum* leaf extract activates TRPA1 channels in a concentration‐dependent manner (EC_50_ ~ 18 μg/mL, efficacy 30%) and also induces their desensitization following AITC response (IC_50_ ~ 60 μg/mL) (Figure 4). Notably, similar effects were observed with *P. cattleianum* fruit extracts that activated TRPA1 with EC_50_ ~ 30 μg/mL but with higher efficacy (~75%) (Figure 4). Additionally, the fruit extract showed TRPA1 channel desensitization at an IC_50_ ~ 47 μg/mL (Figure 4).

**FIGURE 5:**
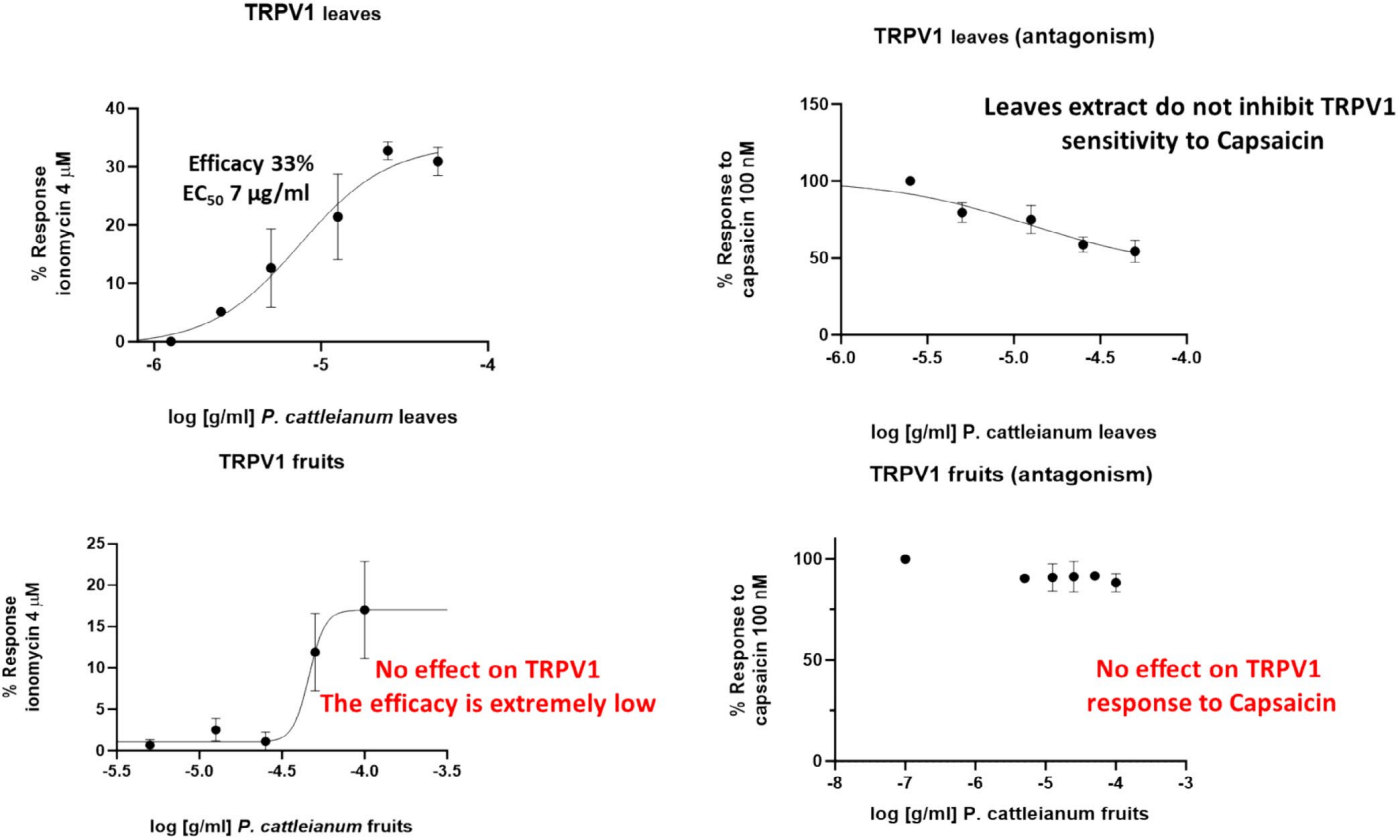
Effect of *P. cattleianum* leaf and fruit extracts on TRPV1 channels. Concentration‐response curves showing the activity of P. Cattle leaves and fruits on TRPV1 channels calculated log(concentration) versus response—variable slope. Ionomycin (4 μM) and Capsaicin (100 nM) were used to calculate the % of *P. cattleianum* response or induced desensitization.

On the other hand, *P. cattleianum* leaf extract activated the TRPV1channel with an EC_50_ of 7 μg/mL and efficacy of 33%. At the same time, no effects were observed on desensitization (Figure 5). Meanwhile, no effects were observed with fruit extracts on TRPV1.

In summary, the different phytochemical profiles of fruit and leaf extracts of *P. cattleianum* resulted in a different biological activities toward the two TRP channels. As mentioned above, the main differences in the profiles of fruits and leaves are found in the phenolic acid and lignan content. Additionally, according to the OPLS‐DA model and log fold change, several discriminant metabolites were found to be characteristic of leaf and fruit extracts, which may justify the significantly stronger inhibition of the TRPA1 sensitivity to AITC of the fruit extract relative to that of the leaves.

TRPA1 is an ion channel involved in various inflammation and pain signaling processes (Abdel‐dayem et al. 2024). Its agonism causes pain, inflammation, and hypersensitivity to thermal and mechanical stimuli. From the chemical point of view, compounds stimulating TRPA1 are commonly electrophiles reacting with cysteine residues of TRPA1 (Macpherson et al. 2007). On the contrary, the analgesic and anti‐inflammatory activity of some compounds results from antagonism or rapid desensitization of TRPA1; this latter is also known as the “paradoxical effect” of agonists (Meotti et al. 2014; Ruparel et al. 2011). Agonist‐dependent desensitization of those ion channels thus results from prolonged exposure to agonists, which induces inactivation, and in some circumstances internalization, of TRPA1 channels (Sanz‐Salvador et al. 2012). Cannabinoids can serve as an example of compounds that exert peripheral antihyperalgesia through functional desensitization of capsaicin and mustard oil responses via activation of the TRPA1 channel on sensory neurons (Akopian et al. 2008).

To the best of the authors' knowledge, this is the first time that *P. cattleianum* has been found to regulate TRP ion channels activity, particularly TRPA1. However, it has been proved that natural compounds are able to modulate TRPA1 channels (Meotti et al. 2014). For example, gallic acid showed relevant antinociceptive effects as a TRPA1 antagonist (Trevisan et al. 2014). Caffeic acid showed anti‐pruritic activity and demonstrated anti‐itching effects by inhibiting multiple mechanisms in HEK 293 cells and mice models, that is, blockade of TRPA1 ion channels (Pradhananga and Shim 2015). Anti‐inflammatory and anti‐nociceptive diterpene carnosol from *Salvia* species was proved to possess an agonistic effect on TRPA1 ion channels in HEK 293 cells. Its activity may result from desensitization of TRPA1 (Zhai et al. 2014). Curcumin, a polyphenolic and electrophilic compound, also activates and desensitizes TRPA1 (Leamy et al. 2011).

Downregulation of TRPV1 receptors by the lignan magnolol was proposed as a possible mechanism of pain and inflammatory alleviation in the burn pain model of mice (Khalid et al. 2019). Gomisin A, a lignan of *Schisandra chinensis*, was also proven to interact with TRPV1 in molecular docking and in vitro studies (Lee et al. 2017). Additionally, the oxygenated monoterpene, safranal (a major compound in saffron) showed dual and selective action on human TRPA1 channels with both activation and further amelioration of desensitization caused by receptor agonists, thus mediating the effect of saffron in pain models in humans and animals (Li Puma et al. 2019).

The electrophilic nature of *P. cattleianum* compounds may contribute to their activity through TRPA1 ion channels. These compounds are more abundant in fruit extracts, probably from phenolic acids and flavonoids. They showed a selective activity on TRPA1; however, no activity on TRPV1 ion channels. Selectivity for TRPA1 channels may be crucial for obtaining an agent with fewer side effects compared to non‐selective compounds, also affecting TRPV1 channels, like locomotor, cardiovascular, or body temperature side effects (Chen et al. 2011).

### 3.4 Molecular Docking

Molecular docking unveiled the characteristics of the site responsible for the compounds' binding affinity and how they interact with the channel. Remarkably, it is widely used to investigate the interactions of natural products with different protein targets (Lekmine et al. 2023). The agonist site lies between two chains with a decent expanse for binding suffused with variable types of amino acids. To perform a molecular docking study of *Psidium cattelianum* phytochemicals, certain compounds were selected (5 in each extract) that follow the criterion of having VIP (≥ 1.2) and Log Fold Change (Log fold change ≥ 2 or ≤ 0.5) from OPLS‐DA analysis and Log Fold change analyses. Thus, they could be considered enriched and discriminant metabolites in their respective extracts. A List of the compounds is presented in Table 3.

**TABLE 3:** List of compounds with VIP (≥ 1.2) and Log fold change (Log fold change ≥ 2 or Log fold change ≤ 0.5) selected for molecular docking.

Compound name	VIP	Fold change	Regulation
Malvidin 3‐*O*‐(6″‐acetyl‐galactoside)	1.266513	22.765263	Up
Theaflavin 3,3'‐*O*‐digallate	1.266599	21.320042	Up
Delphinidin 3‐*O*‐(6″‐acetyl‐galactoside)	1.266607	20.898392	Up
Galloyl glucose	1.266543	20.65476	Up
Petunidin 3‐*O*‐(6″‐acetyl‐galactoside)	1.263884	20.4436	Up
Theaflavin 3‐*O*‐gallate	1.266568	−22.026258	Down
Piceatannol	1.266552	−21.439594	Down
Narirutin 4'‐*O*‐glucoside	1.266513	−21.34645	Down
Quercetin 3‐*O*‐xylosyl‐rutinoside	1.26645	−21.146275	Down
Pelargonidin 3‐*O*‐glucosyl‐rutinoside	1.266465	−20.564224	Down

**FIGURE 6:**
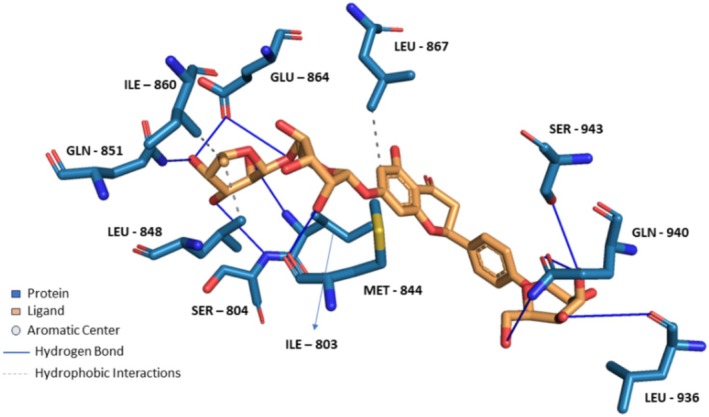
Docking of Theaflavin 3,3'‐*O*‐digallate to TRPA1 ion channel (PDB ID: 6X2J) analyzed on Protein Ligand Interaction Profiler website and visualized on Pymol software.

Compounds enriched in the fruit extract, as represented by theaflavin 3‐*O*‐gallate, piceatannol, narirutin 4'‐*O*‐glucoside, quercetin 3‐*O*‐xylosyl‐rutinoside, and pelargonidin 3‐*O*‐glucosyl‐rutinoside, were examined and were found to bind efficiently to the cavity, as represented in Table 4 with notably high binding affinity represented as respective S scores. Moreover, the molecules interact with the receptor with a versatile set of interactions (including hydrogen bonds, П‐Cation, П‐П Stacking, and hydrophobic interactions) and mostly fit to the site in a comparable manner to the co‐crystallized ligand (Table 4). Noticeably, the larger the compound, the more interactions it displays, and the better occupation of the binding site it elicits, which augments a robust felicitous affinity to the protein. For instance, piceatannol is the smallest among the structures, possessing the lowest S score of −5.36 Kcal/mol and the least number of interactions. On the other hand, the glycosides of narirutin, quercetin, and pelargonidin, which are larger in size, have more interactions and better S scores of around −8 Kcal/mol. Reasonably, the highest affinity goes to theaflavin 3,3'‐*O*‐digallate, a leaf‐extracted molecule with a molecular weight of 868.7 g/mol, which excels all other compounds. It displayed an S score of −9.27 Kcal/mol by virtue of forming a set of 5 hydrogen bonds and 6 hydrophobic interactions, 1 П–cation, and 1 П–stacking interaction (Figure 6). Yet, overall, compounds enriched in the leaf extract bind with lower interactions and lower S scores of around −7 Kcal/mol compared to fruit extract‐enriched ones (Table S3). Also, docking results show that the highest binding molecule is narirutin‐4'‐*O*‐glucoside, with a total number of interactions of 10 hydrogen bonds and 3 hydrophobic interactions. The compounds investigated from the fruit extract tend to be more fitted and more bound to the receptor, which aligns with their better efficacy in the TRPA1 Assay.

**TABLE 4:** Docking of Fruit extract compounds by MOE‐v.2019.01. All compounds were analyzed through the PLIP website, and poses were captured using Pymol software.

Compound name	3D docking pose	S score (Kcal/mol)	No. and types of interactions
Theaflavin 3‐*O*‐gallate	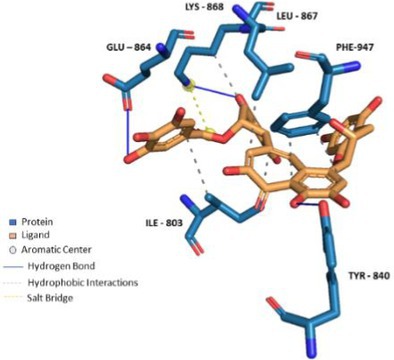	−8.19	3 Hydrogen Bonds. 6 Hydrophobic Interactions. 1 Salt Bridge.
Piceatannol	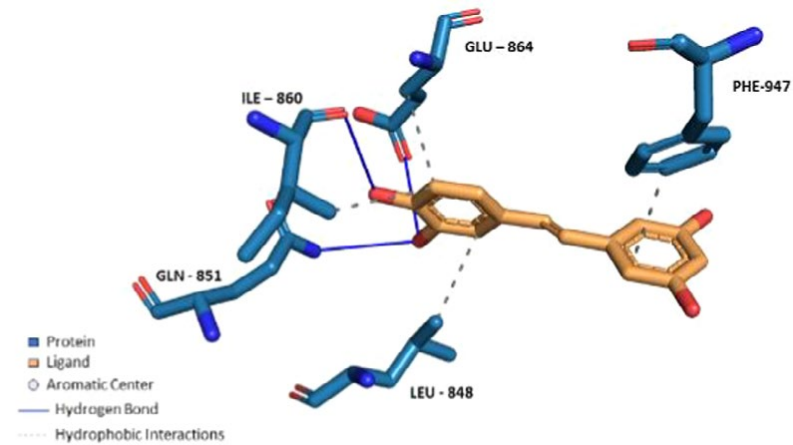	−5.36	3 Hydrogen Bonds. 4 Hydrophobic Interactions.
Narirutin 4'‐*O*‐glucoside	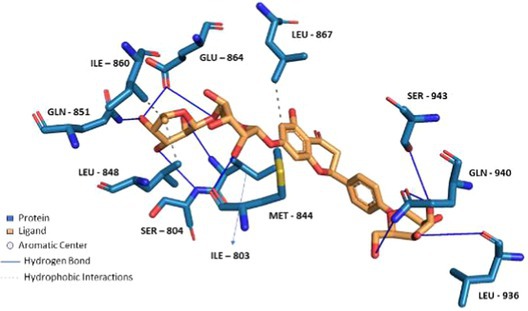	−8.20	10 Hydrogen Bonds. 3 Hydrophobic Interactions.
Quercetin 3‐*O*‐xylosyl‐rutinoside	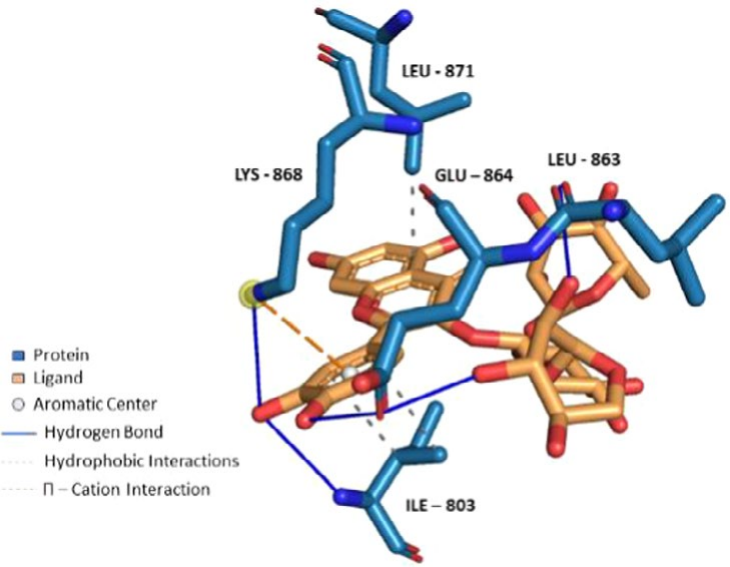	−8.92	5 Hydrogen Bonds. 3 Hydrophobic Interactions 1 П—Cationic Interaction
Pelargonidin 3‐*O*‐glucosyl‐rutinoside	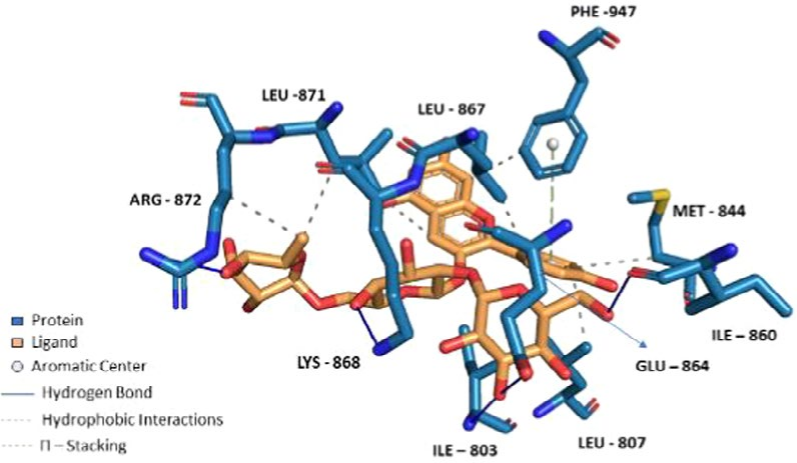	−8.61	5 Hydrogen Bonds. 7 Hydrophobic Interactions. 1 П—Stacking

**FIGURE 7:**
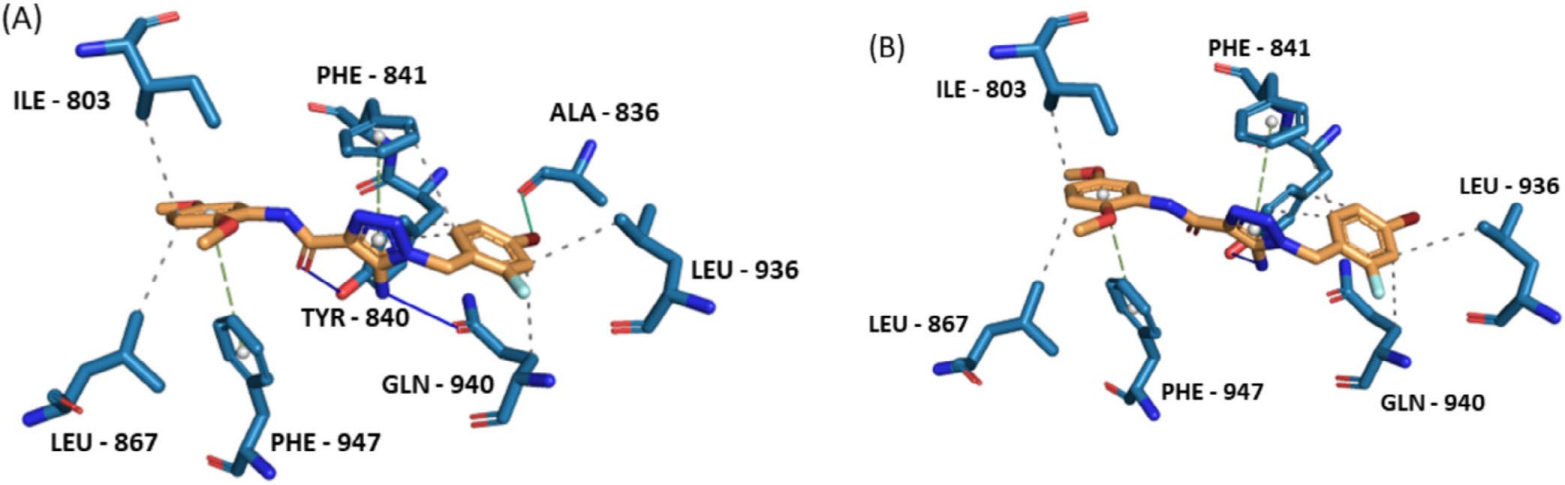
Agonist co‐crystallized with TRPA1 (PDB ID: 6X2J). (A) interactions of the co‐crystalized ligand. (B) interactions of the re‐docked co‐crystallized ligand using MOE—v.2019.01.

To validate the docking protocol and the ability of the docking tool to re‐produce the docking pose and interactions of the co‐crystallized ligand of the crystal structure (PDB ID: 6X2J), it was docked applying default parameters using MOE‐v.2019.01. The docked ligand demonstrated an RMSD value of 0.2583 Å^2^ and an S score of −7.60 Kcal/mol. Figure 7 demonstrates the re‐docking results compared to the original interactions observed for the co‐crystallized ligand. Figure 7A displays the co‐crystallized ligand interactions analyzed by the PLIP website. The co‐crystallized agonist makes 5 interactions with the protein (2 hydrogen bonds with TYR‐840, one hydrogen bond with SER‐943, one PI—Stacking with PHE‐947 and one PI—Stacking with PHE‐841). Figure 7B shows the re‐docked ligand interactions as visualized by the PLIP tool. Thus, it can be concluded that the docking tool and protocol can be used to predict the binding poses and interactions of the test compounds.

Few studies dealt with the molecular interactions of the natural agonists for TRPA1 channels. For instance, the phenolic monoterpenes, thymol and carvacrol, showed hydrogen bonding and hydrophobic interactions with the amino acid residues of TRPA1 receptors. This further advocates for their in vitro studied potential as agonist molecules for these channels (Ghosh et al. 2020).

According to the results, catechins as represented by theaflavin 3,3'‐*O*‐digallate and theaflavin 3‐*O*‐gallate showed significant interactions with TRPA1 on the molecular level; this is in line with previous reports on the potential of tea catechins such as epigallocatechin gallate (EGCG) to activate TRPA1 in STC‐1 cell lines (Kurogi et al. 2012). Interestingly, Yamazaki et al. (2014) reported that theaflavin 3,3'‐*O*‐digallate and theaflavin 3‐*O*‐gallate increased [Ca^2+^]_i_ at micromolar levels in human bitter taste receptors in HEK293 cells, and they denoted that this effect could be attributed to the presence of galloyl moieties. These findings agree with the molecular docking study's better performance observed for the di‐galloyl compound (theaflavin 3,3'‐*O*‐digallate).

The activity of flavonoid glycosides on TRPA1 channels was also previously observed. For instance, liquiritin, the 4'‐*O*‐glucoside of liquiritigenin, was proved to be an inhibitor of TRPA1 and TRPV1 channels in a mouse acute lung injury model induced by lipopolysaccharide (LPS) (Liu et al. 2020). However, a study by Sanechika et al. (2021) indicated the agonistic effect of flavonoid aglycones on TRPA1, while none of the flavonoid glycosides displayed this kind of activity. Quercetin, another example of flavonoids, is also a possible ligand of the TRPA1 receptor, which does not affect TRPV1 (Nakamura et al. 2016). Those reports indicate the need for further research on the effect of these compounds on TRP channels.

### 3.5 Molecular Dynamics Simulation

Molecular Dynamics (MD) is a computational simulation that models the time‐dependent behavior of interacting atoms by solving their motion equations according to Newton's laws. This approach enables the calculation of particle trajectories within a multi‐dimensional phase space, facilitating the analysis of energy and behavior in systems with numerous atoms (Kumar and Ojha 2023).

**FIGURE 8:**
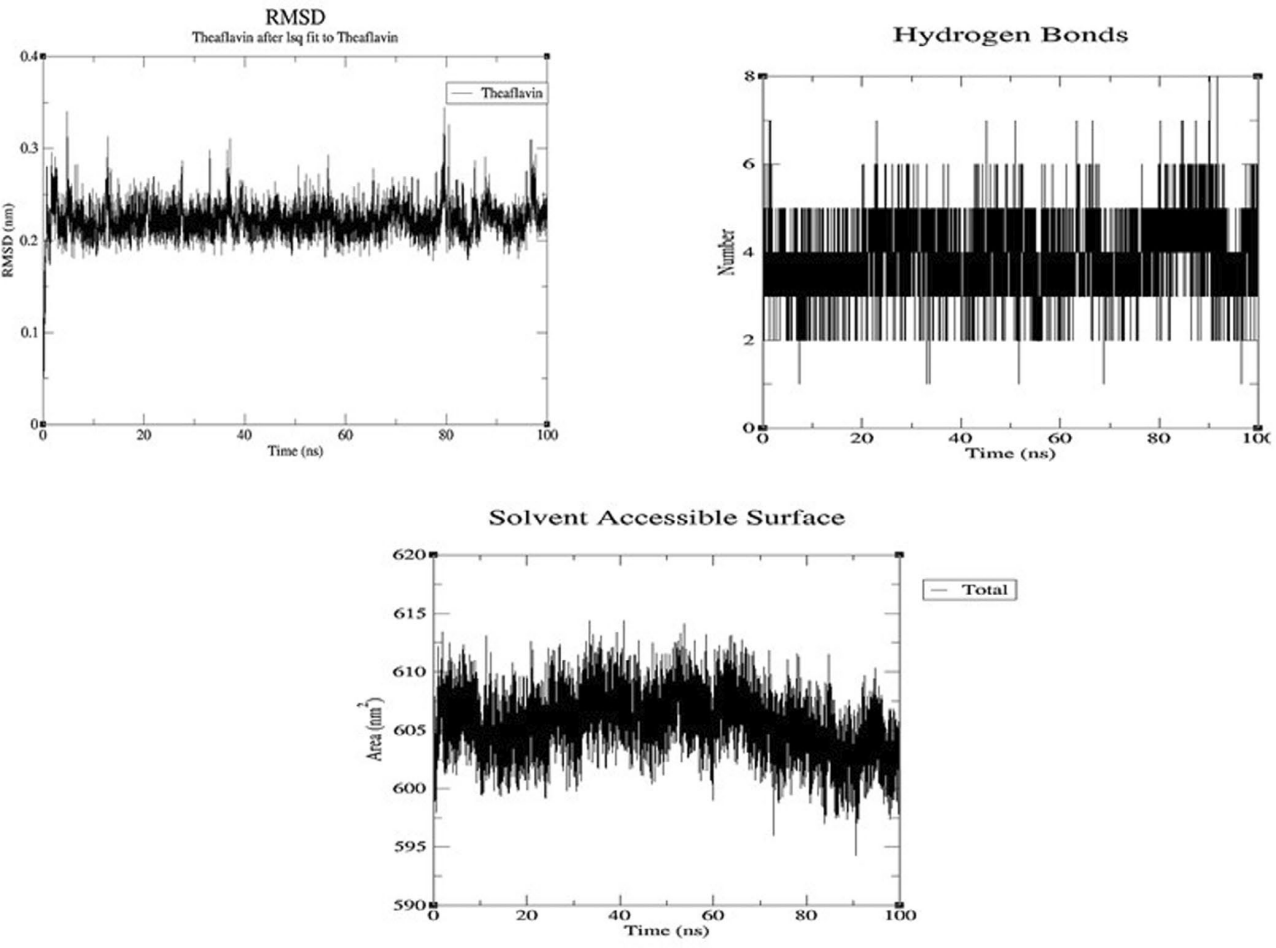
Molecular dynamics results from a 100 ns simulation of TRAP1‐theaflavin 3,3'‐*O*‐digallate complex: (upper left panel) ligand RMSD; (upper right panel) H‐Bonds formation; and (lower panel) SASA.

To gain more insights about the stability and compactness of the binding between theaflavin 3,3'‐*O*‐digallate (A representative compound with the highest binding score of *P. cattleianum* leaf extract) to the TRPA1 ion channel, MD simulation was conducted and analyzed by three main indicators, which are the Root Mean Square Deviation (RMSD), Number of Hydrogen Bonds, and the Solvent Accessible Surface Area (SASA) (Figure 8). The high potentials of the theaflavin 3,3'‐*O*‐digallate conformed with its RMSD, where it demonstrated a fluctuation pattern with an average oscillation value of 0.18 nm during the whole simulation. In addition, the remarkable stability was also indicated by the number of H‐bonds formed throughout the simulation with an average of 3–6 H‐bonds. The high compactness of the interaction was also highlighted with the minor SASA fluctuation ranging between 600 and 615 nm^2^ throughout the whole 100 ns simulation. These results affirm the high binding affinity and stability between theaflavin 3,3'‐*O*‐digallate and TRPA1.

## 4 Conclusion

An untargeted metabolomics study was performed to find the encompassed phytochemicals in the fruit and leaf extracts of *Psidium cattleianum*. This enabled the annotation of 469 compounds belonging to different phenolic subclasses with a predominance of flavonoids and phenolic acids. Moreover, multivariate data analysis led to the identification of discriminant metabolites across both matrices. The fruit extract exhibited significant efficacy against TRPA1 channels, and molecular docking further augmented the biological interactions with the receptors, which support the molecular findings. Interestingly, this is the first study investigating the effect of *Psidium* extracts on TRPA1 and TRPV1 channels. The current study successfully gives deep insights into the phytochemical signature of *P. cattleianum* extracts and recommends further exploitation of the fruit extract in pharmaceutical products to alleviate pain.

## Author Contributions

**Leilei Zhang:** data curation (equal), formal analysis (equal), investigation (equal), methodology (equal), software (equal), validation (equal), writing – original draft (equal), writing – review and editing (equal). **Fabio Arturo Iannotti:** formal analysis (equal), investigation (equal), methodology (equal), validation (equal), visualization (equal), writing – original draft (equal), writing – review and editing (equal). **Fatema R. Saber:** conceptualization (equal), investigation (equal), writing – original draft (equal), writing – review and editing (equal). **Reem K. Arafa:** data curation (equal), formal analysis (equal), software (equal), validation (equal), visualization (equal), writing – original draft (equal). **Aniello Schiano Moriello:** formal analysis (equal), investigation (equal). **Rasha A. Rasle:** data curation (equal), formal analysis (equal), software (equal), validation (equal), visualization (equal), writing – original draft (equal). **Anton Soria‐Lopez:** investigation (equal), visualization (equal), writing – original draft (equal), writing – review and editing (equal). **Sara G. Abd EL‐Gawwad:** software (equal), validation (equal), visualization (equal), writing – original draft (equal). **Gabriele Rocchetti:** investigation (equal), writing – review and editing (equal). **Paz Otero:** investigation (equal), visualization (equal), writing – original draft (equal), writing – review and editing (equal). **Łukasz Kulinowski:** investigation (equal), writing – original draft (equal), writing – review and editing (equal). **Krystyna Skalicka‐Woźniak:** supervision (equal), writing – review and editing (equal). **Luigi Lucini:** conceptualization (equal), funding acquisition (equal), supervision (equal), writing – review and editing (equal). **Jesus Simal‐Gandara:** supervision (equal), writing – review and editing (equal).

## Ethics Statement

Ethical approval was not sought for the present study because the work did not involve studies using animal experimentation.

## Conflicts of Interest

The authors declare no conflicts of interest.

## Supporting information


Data S1.

## Data Availability

The data that support the findings of this study are available from the corresponding authors upon reasonable request.
