# Supplementary material for: The Phenolic Signature of Psidium cattleianum Fruits and Leaves Modulates TRPV1 and TRPA1 Transient Receptor Potential Channels: A Metabolomics, In Vitro, and In Silico Study
Source: Food Sci Nutr. 2025 Mar 24;13(4):e70075. doi: 10.1002/fsn3.70075 (PMC11931593; doi:10.1002/fsn3.70075)
Supplement: Supplementary file 1 — Data S1. [file FSN3-13-e70075-s001.docx]

**The Phenolic signature of *Psidium cattleianum* Fruits and Leaves Modulates TRPV1 and TRPA1 Transient Receptor Potential Channels: A Metabolomics, In Vitro, and In Silico Study**

**Leilei Zhang^1$^, Fabio Arturo Iannotti^2$^, Fatema R. Saber^3*^, Reem K. Arafa^4, 5^, Aniello Schiano Moriello^2^, Rasha A. Rasle^4, 5^, Anton Soria-Lopez^6^, Sara G. Abd EL-Gawwad^4, 5^, Gabriele Rocchetti^7^, Paz Otero^8*^, Łukasz Kulinowski^9^, Krystyna Skalicka-Woźniak^9^, Luigi Lucini^1^, Jesus Simal-Gandara^8,10*^**

^1^ Department for Sustainable Food Process, Università Cattolica del Sacro Cuore, Via Emilia Parmense 84,29122 Piacenza, Italy

^2^Institute of Biomolecular Chemistry (ICB); National Research Council (CNR), viale Campi Flegrei 34 80078 Pozzuoli (NA)

^3^Pharmacognosy Department, Faculty of Pharmacy, Cairo University, 11562, Cairo, Egypt

^4^Drug Design and Discovery Lab, Helmy Institute for Medical Sciences, Zewail City of Science and Technology, Giza, Egypt, 12578

^5^Biomedical Sciences Program, University of Science and Technology, Zewail City of Science and Technology, Giza, Egypt, 12578

^6^Department of Physical Chemistry, Faculty of Sciences, Universidade de Vigo, Ourense 32004, Spain

^7^ Department of Animal Science, Food and Nutrition, Università Cattolica del Sacro Cuore, Via Emilia Parmense 84, 29122 Piacenza, Italy

^8^ Nutrition and Bromatology Group, Analytical Chemistry and Food Science Department, Faculty of Science, E32004 Ourense, Spain

^9^Department of Natural Products Chemistry, Medical University of Lublin, Lublin 20-093, Poland

^10^CISPAC, Fontan Building, City of Culture, E15707 Santiago de Compostela, <https://cispac.gal/>

^$^ The authors equally contributed to the manuscript

* Corresponding authors: [Fatema.saber@pharma.cu.edu.eg](mailto:Fatema.saber@pharma.cu.edu.eg), luigi.lucini@unicatt.it, [jsimal@uvigo.es](mailto:jsimal@uvigo.es)

**^#^**The authors share the seniorship.

**SUPPLEMENTARY TEXT**

**Untargeted UHPLC-ESI/QTOF profiling**

The phenolic compounds are profiled through a 1290 UHPLC chromatograph, equipped with a binary pump and a Dual Electrospray JetStream ionization source, coupled to a hybrid QTOF mass analyzer (G6550 mass spectrometer, Agilent Technologies, Santa Clara, CA, USA). The chromatographic separation was achieved under a water-acetonitrile, using 0.1 % (*v/v*) formic acid as phase modifier (both LC-MS grade, from Sigma-Aldrich, Milan, Italy), gradient elution (6–94 % acetonitrile in 32 min), flow rate of 0.2 mL/min and injection volume of 6 μL -Agilent Poroshell 120 PFP column (100 mm × 2.1 i.d., 1.9 μm particle size). The QTOF mass analyzer operated in positive mode (ESI+) for both MS and MS/MS acquisition with nitrogen as both sheath gas (12 L/min and 315 ◦C) and drying gas (14 L/min and 250 ◦C). The nebulizer pressure was 45 psi, the nozzle voltage was 350 V, and the capillary voltage was 4.0 kV. For MS acquisition, the full scan mode was performed within the range of *m*/*z* 100–1200 (1 spectra/s), with a mass resolution of 30,000 full width at half maximum (FWHM), *m*/*z* = 200. The data-dependent mode was performed for precursor fragmentation (10, 20, and 40 eV) and acquisition of MS/MS data from QC samples, with a mass resolution of 30,000 (FWHM), selecting 8 precursors per cycle (1 Hz, *m*/*z* 80–1200, positive polarity, and active exclusion after 2 spectra). The Processing of the chromatograms was carried out using the MassHunter Qualitative Analysis software (version B.06.00, Agilent Technologies).

The raw mass features were processed using Profinder B.07 (Agilent Technologies) software, based on the “find-by-formula” algorithm. Compound annotation was recursively achieved, following mass and retention time alignment, against the database Phenol-Explorer (Phenol-Explorer 3.6; http://phenol-explorer.eu/, accessed July 2, 2023). The annotation was based on the isotopic profile of each molecular feature detected (consisting of a monoisotopic mass, isotope spacing, and ratio combination, with a mass accuracy of 5 ppm). Data filtering was used to remove the features not detected in at least 75 % of replications per each tested group. According to the Metabolomics Standards Initiative (MSI), confidence Level 2 of identification (i.e., putatively annotated compounds, COSMOS standards in metabolomics) [1].

Subsequently, compounds were assigned to classes and subclasses and then quantified with single pure standards analyzed by the same method. The standards used were representative of the following classes of bioactive compounds: anthocyanins (cyanidin), flavanols and flavonols (catechin), flavones (luteolin), phenolic acids (ferulic acid), lignans (sesamin), stilbenes (resveratrol) and low molecular weight phenolics (tyrosols). Results are expressed as mg equivalents/g dried extract.

**SUPPLEMENTARY TABLE**

**Table S3.** Docking of leaf extract molecules to the channel (PDB ID: 6X2J) at the agonist site by MOE-v.2019.01.

| **Compound Name** | **3D Docking pose** | **S Score**  **(Kcal/mol)** | **No. and types of interactions** |
| --- | --- | --- | --- |
| **Malvidin 3-*O*-(6''-acetyl-galactoside)** | 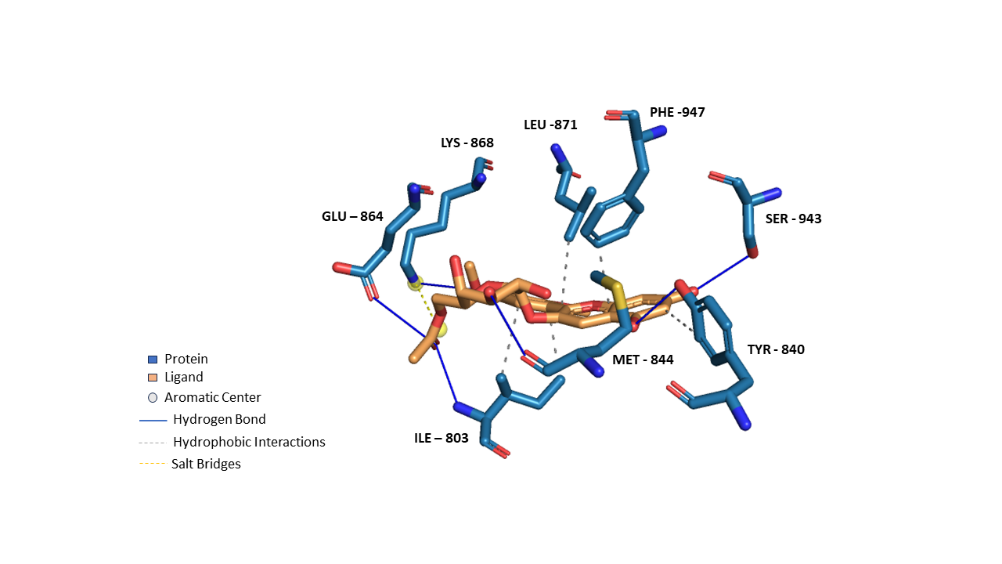 | -7.68 | - 6 Hydrogen Bonds. - 5 Hydrophobic   Interactions.   - 1 Salt Bridge |
| **Theaflavin 3, 3'-*O*-digallate** | 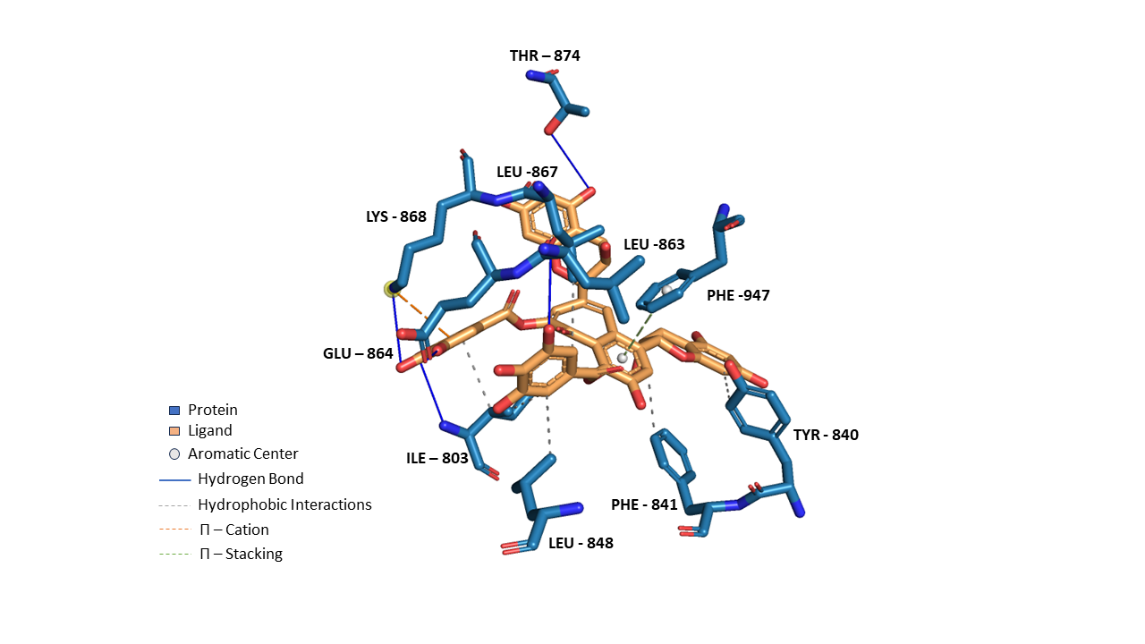 | -9.27 | - 5 Hydrogen Bonds. - 6 Hydrophobic Interactions. - 1 П – Cation - 1 П – Stacking |
| **Delphinidin 3-*O*-(6''-acetyl-galactoside)** | 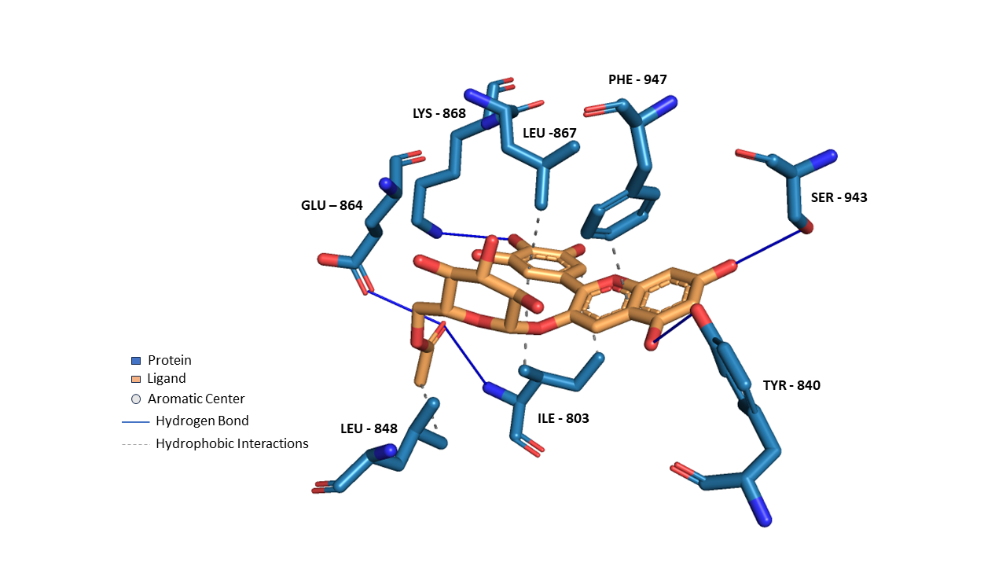 | -7.72 | - 5 Hydrogen Bonds. - 4 Hydrophobic Interactions. |
| **Galloyl Glucose** | 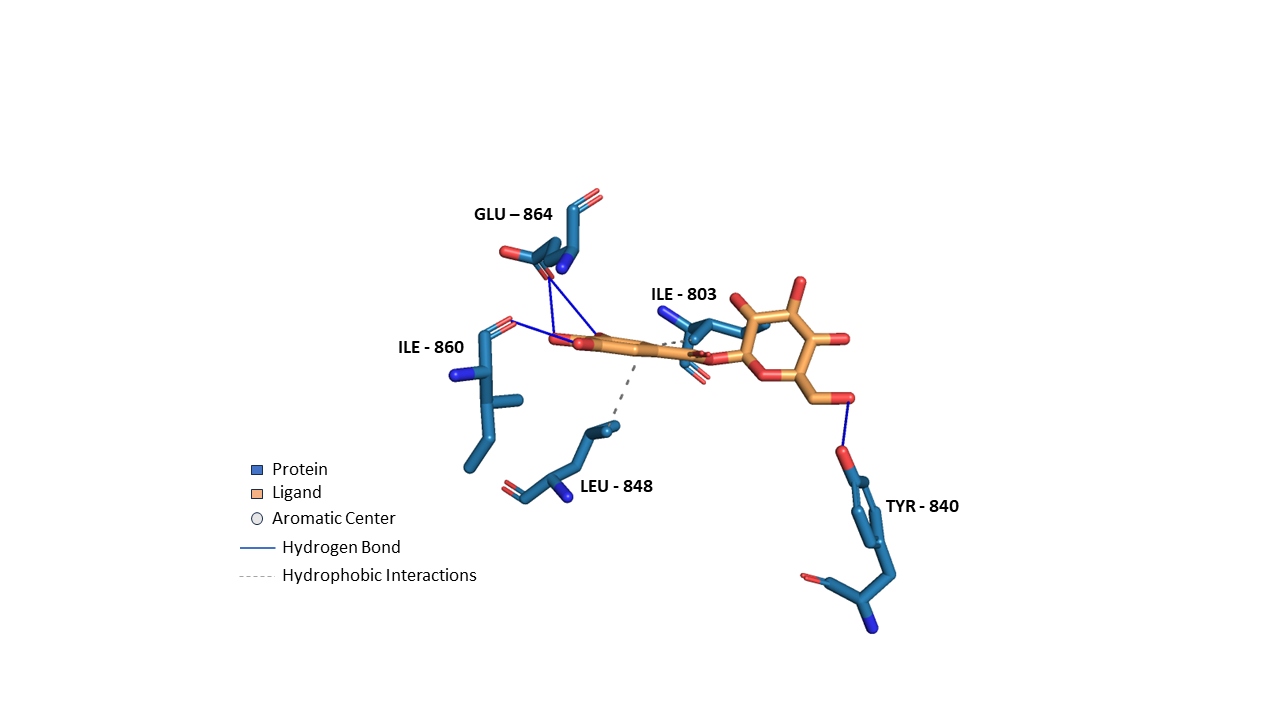 | -6.05 | - 4 Hydrogen Bonds. - 2 Hydrophobic Interactions. |
| **Petunidin 3-*O*-(6''-acetyl-galactoside)** | 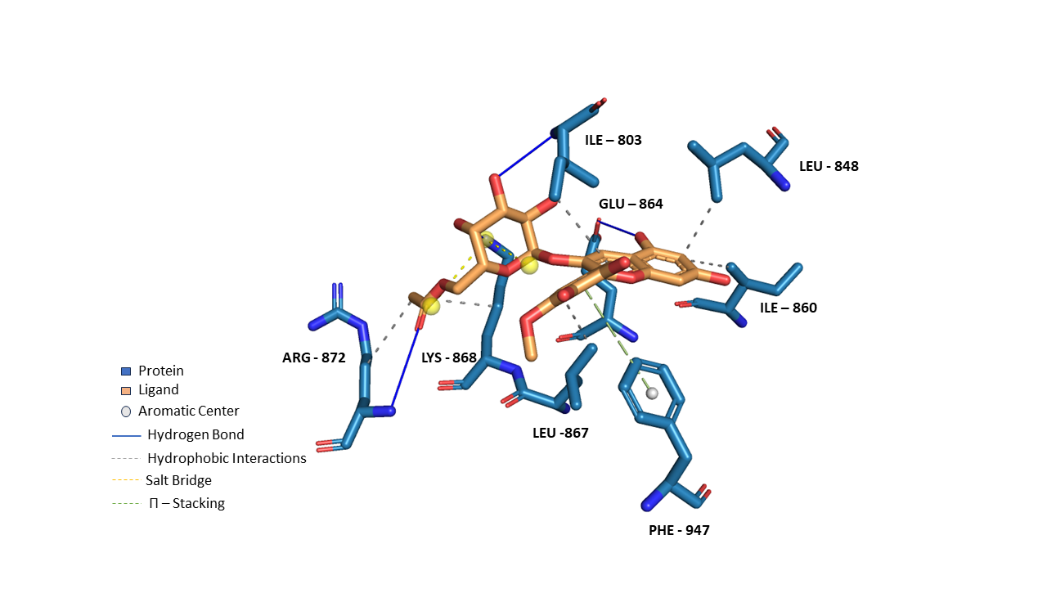 | -7.17 | - 4 Hydrogen Bonds. - 6 Hydrophobic Interactions. - 2 Salt Bridges - 1 П – Stacking |

1. Salek RM, Neumann S, Schober D, Hummel J, Billiau K, Kopka J, Correa E, Reijmers T, Rosato A, Tenori L: **COordination of Standards in MetabOlomicS (COSMOS): facilitating integrated metabolomics data access**. *Metabolomics* 2015, **11**:1587-1597.
